# Worldwide demand for congenital heart surgery programs and surgeons

**DOI:** 10.1186/1749-8090-8-S1-O304

**Published:** 2013-09-11

**Authors:** M Cardarelli, F Molloy, I Polivenok, H Rodriguez Saldaña, W Novick

**Affiliations:** 1 International Children Heart Foundation, Memphis, TN, USA; 2 Institute of General and Urgent Surgery, AMS of Ukraine, Kharkov, Ukraine

## Background

Congenital heart disease (CHD) programs are essential in the prevention of early and late deaths. Vital to program building efforts is the availability of properly trained local cardiac surgeons. We developed an ideal Surgical-CHD-Incidence/Surgeon Ratio and ranked countries accordingly.

## Methods

We used publicly available data (CTSnet, EACTS, WHO) and personal communications. We calculated the yearly incidence of Severe/Moderate CHD births for each country (surgical CHD incidence). An ideal ratio was developed based on known ratios in developed nations.

## Results

Worldwide, 3,054 surgeons self-describe as qualified to perform pediatric cardiac surgery on CTSnet. Extrapolating from North American surveys, it is likely that only 35% of them are trained to treat complex forms of CHD. Ideal Surgical-CHD-Incidence/Surgeon ratio was set at 140 (highest ratio with documented coverage of all surgical CHD). Accordingly, countries were ranked into 3 groups: Group I: 58 countries with 94,459 new surgical patients/year. With a Surgical-CHD-Incidence/Surgeon ratio < 140, given enough resources, most children born in these societies should have a chance of timely surgical treatment. Group II: 53 countries with 591,237 new surgical patients/year. With a Surgical-CHD-Incidence/Surgeon ratio >140 and limited ability to provide adequate surgical care. Group III: 69 countries with 124,259 new surgical patients/year. (Includes 16 smaller or insular countries with less than 100 new surgical CHD births/year). No pediatric cardiac surgeon is available in these countries.

## Conclusions

While a ratio < 140 does not guarantees treatment of all children born with surgical CHD, at ratios >140 coverage is limited. Based on current estimates, there is a deficit of between 2,500- and 4,500 congenital heart surgeons worldwide. We believe that international humanitarian organizations should concentrate CHD program building efforts primarily on Group II and failing Group I countries.

